# Lethal effects of allyl isothiocyanate on the egg masses of flighted spongy moth complex *Lymantria dispar japonica* (Lepidoptera: Erebidae)

**DOI:** 10.1002/ps.8981

**Published:** 2025-06-18

**Authors:** Shunya Murase, Keisuke Matsui, Hiromi Asai, Daisuke Hayasaka, Takuo Sawahata

**Affiliations:** ^1^ Graduate School of Agriculture, Kindai University Nara Japan; ^2^ PRD Co., Ltd. Osaka Japan; ^3^ Faculty of Agriculture, Kindai University Nara Japan

**Keywords:** biological invasion, ovicide, pest management, susceptibility, volatile organic compounds, forest pest

## Abstract

**BACKGROUND:**

The flighted spongy moth complex (FSMC) lays egg masses not only on host plants but also various artificial structures, such as, cargo, vehicles, and ships. Thus, preventing the transmission of these egg masses is an international challenge. Their eggs are covered with thick hair covering that act as a barrier to insecticides thus making them ineffective. A volatile gas may be able to carry toxic substances past this barrier to the eggs. In this study, we evaluated the insecticidal effect of the highly volatile allyl isothiocyanate (AITC) on *Lymantria dispar* egg masses and compared it with that of two conventional insecticides containing permethrin, fenpropathrin, and clothianidin.

**RESULTS:**

In the two conventional insecticide treatments, approximately 50% of the eggs survived when the hairy covering was not removed. In contrast, all eggs, with or without hairy covering, did not hatch after AITC treatment. The LC_50_ and LC_90_ values of AITC against them were 37.3 ± 5.4 and 100.9 ± 11.8 ppm, respectively. Our results show that AITC at 35000 ppm can eradicate FSMC egg masses.

**CONCLUSION:**

AITC has a lethal effect against FSMC egg masses and may be applied for the extirpation of eggs/egg masses of various other organisms, besides FSMC. © 2025 The Author(s). *Pest Management Science* published by John Wiley & Sons Ltd on behalf of Society of Chemical Industry.

## Graphical abstract

This study showed that AITC substance was lethal effect to *Lymantria dispar* japonica egg mass, and our results show a significant decline in egg hatching rate with increasing AITC concentrations.

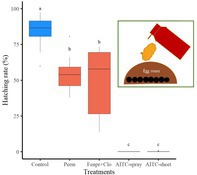

## 1 INTRODUCTION

In recent years, invasive species introduced *via* containers of cargo and ballast water have become a global problem.^1^ Invasive species have adverse effects on ecosystems and human health, and large amounts of money are spent on their management and control.^2^ Previous research indicates that preventing the introduction of these invaders is an effective low‐cost control option and warrants the highest priority. Therefore, it is essential to develop cost‐effective control methods to prevent the spread of various invasive species.^1^

Among invasive species, the spread of various herbivorous Lepidoptera species has been particularly affected by the increased transportation and trade of stored plant products.^3^ *Lymantria dispar* Linnaeus (Lepidoptera: Erebidae) (spongy moth) is widely distributed in temperate Asia, including Japan, and in Europe, North Africa, and eastern North America.^4^ This species has periodically been reported to cause outbreaks,^5^ and its larvae feed on over 300 species of herbaceous and woody plants, including broadleaf trees and conifers.^6^ In general, adult *L. dispar* females lay a single egg mass^7^ consisting of 300–1000 eggs, which are covered by thick hair.^4^ *L. dispar* larvae that hatch from egg masses can reach and/or disperse to host plants by ballooning behavior (i.e. moving with the wind).^4^ In North America, the degradation of tree stands during mass outbreaks of *L. dispar* larvae resulted in a reduction in forest recreation and timber values.^7^, ^8^ The United States Department of Agriculture (USDA) estimated that this species has caused an average of $30 million of economic damage over the past two decades.^9^

*L. dispar* has been divided into three subspecies based on genetic differences.^10^ The European spongy moth *Lymantria dispar dispar* is distributed in Europe and was introduced to North America.^10^ The flighted spongy moth complex (FSMC) includes *Lymantria dispar asiatica* and *Lymantria dispar japonica*, which are widely distributed across Asia.^10^ Invasive *L. dispar* has been identified as one of the 100 most invasive species globally.^1^ Previous researchers devised these two groups based on the presence or absence of female flight ability. The European spongy moth female lacks flight capability, whereas FSMC females can fly a distance of approximately 1 km and have dispersal capacity.^10^, ^11^ Among these subspecies, FSMC is considered to have a particularly significant impact on the habitats of invaded regions.^12^, ^13^

Owing to their flight ability, FSMC egg masses are found not only on host plants but also on various artificial structures such as eaves, telephone poles, and cracks or crevices on rock outcrops.^4^ Furthermore, FSMC spends approximately 9 months of its life cycle in the egg stage.^4^, ^7^ When FSMC female laid egg masses on cargo, vehicles, and ships, the egg masses easily transmit long distances.^14^, ^15^, ^16^ Therefore, the egg stage could be considered the best developmental stage to target to reduce the risk of invasion from high‐risk points such as seaports and airports. Indeed, when vessels enter non‐invaded areas from native or known FSMC habitats, they must provide a certificate confirming that they are not carrying FSMC egg masses.^14^ However, unintentional FSMC transportation in connection with trade can occur, so it is important to conduct an effective eradication process to remove FSMC egg masses attached to or contained in the cargo.

Currently, control measures for *L. dispar* egg masses include physical removal and the direct application of soybean oil to suffocate the eggs.^17^ The very low efficacy of chemical insecticides against *L. dispar* eggs may be due to the hairy covering on the egg masses, which prevents the insecticide from penetrating the eggs. Consequently, there is limited information on the effectiveness of insecticide application for eradicating FSMC egg masses.

Allyl isothiocyanate (AITC, C₄H₅NS) is a natural insecticidal substance found in *Brassica juncea* (L.) Czern. (mustard), *Eutrema japonicum* (Miq.) Koidz. (wasabi), and other cruciferous vegetables and has strong volatility. It is produced when the myrosinase enzyme reacts with glucosinolate sinigrin in the presence of water.^18^ In addition, concerns about residues are relatively low because of their easy decomposition in the atmosphere; thus, AITC is perceived to be safe for both humans and the environment.^19^ Some studies have indicated that AITC has a lethal effect on *Solenopsis invicta* Buren (fire ant) workers^20^ and halts the embryonic development of *Xenopus laevis* Daudin (African clawed frogs).^21^ These studies suggest the adverse effects of AITC on various arthropods, regardless of their life stage (e.g., adult or embryonic).

We hypothesized that AITC can pass through the hairy coverings of FSMC eggs and penetrate the egg surface because of its high volatility and have lethal impact on the hatching rates. Thus, in this study, to evaluate the efficacy of AITC against egg masses of FSMC, two experiments were conducted. Our first aim was to evaluate the protective effect of hairy coverings against insecticides by selecting the systemic chemical clothianidin and broad‐spectrum pyrethroid insecticides permethrin and fenpropathrin, which are effective against FSMC and commonly used in Japan, and comparing them with the volatile chemical AITC. FSMC egg masses were exposed to AITC‐containing products (AITC‐spray and AITC‐sheet) and two other conventional insecticides (permethrin (perm.) and fenpropathrin and clothianidin (fenpr+clo)), and their hatchability rates were compared after a month. Second, a 24‐h (acute toxicity) concentration‐susceptibility test was conducted for AITC in FSMC eggs.

## 2 MATERIALS AND METHODS

### 2.1 Sample collection and treatments

Egg masses of FSMC were collected from two cities, Takato Town (35°83′21.4″ N, 138°06′28.9″ E) and Matsumoto City (36°26′21.1″ N, 137°95′47.8″ E) on 17 March 2021 in Nagano Prefecture, Japan, where an outbreak of the species had occurred in 2020 and 2021. Collected samples were brought back to the laboratory and stored in a refrigerator at 5°C. Most of the collected egg masses were used to compare the lethal effects of existing insecticide products and AITC‐containing products on FSMC eggs. The remaining samples were placed individually in Petri dishes and stored in the laboratory at 25 ± 1°C until hatching. The hatched FSMC larvae were reared using an artificial diet. The artificial diet was prepared by mixing *Morella rubra* Lour. leaf powder with Insecta F‐II (Insect Larva base food ‐ mash type, NOSAN INSECT MATERIALS Inc., Kanagawa): water in 30 g: 20 g: 130 mL.^22^ After rearing, male and female adults were collected and placed in plastic containers (40 cm length × 40 cm width × 60 cm height) to produce egg masses. The removal of the hairy coverings from *L. dispar* egg masses and rearing of larvae followed the methods described by Shimazu,^23^ wherein the hairy covering of the egg masses was removed by placing them in a stocking and vacuuming from outside the fabric.^23^

### 2.2 Effects of AITC and conventional insecticides on hatching suppression of FSMC egg masses

The strength of the hatching suppression effect on FSMC egg masses was compared between AITC and two conventional insecticides: one containing 0.2% permethrin (Kinchor E for Gardening, Sumitomo Chemical Garden Products Inc. Tokyo, Japan) (perm.) and another containing 0.020% fenpropathrin and 0.032% clothianidin (systemic neonicotinoid insecticides) (Benica Kemushi aerosol, Sumitomo Chemical Garden Products Inc.) (fenpr+clo). The treatments tested were as follows: control (no insecticide), AITC‐spray (4% AITC, 0.43 g/s; Wasabi‐spray S (https://www.wasap.jp/product.php), PRD Inc., Osaka, Japan), AITC‐sheet (1000 ppm of AITC per 1 cm^2^; Wasabi‐sheet (https://www.wasap.jp/product.php), PRD Inc.), and two kinds of conventional insecticide sprays (perm.; 0.68 g/s and fenpr+clo; 0.3 g/s).

Each insecticide had a lethal effect on 200 FSMC eggs whose coats had been removed in the preliminary tests (Table 1). The preliminary test was conducted under the same conditions as the formal test. In the formal tests, 15 egg masses of FSMC were used in each treatment. All treatments were carried out using the spraying methods employed usually for permethrin, fenpropathrin, and clothianidin. These three treatments were sprayed on FSMC egg masses for 1 s from a distance of 30 cm in the outdoor campus of the Faculty of Agriculture, Kindai University, Nara, Japan (34°40′ 19.5″ N, 135°43′ 56.0″ E). Only the AITC‐spray treatments were left outdoors for 24 h to allow complete volatilization. After each treatment (control, AITC‐spray, and two other insecticide sprays), the egg masses were stored at a constant temperature of 25°C for 1 month, and their hatchability was measured. To check the efficacy of AITC sheets, an AITC sheet (4 cm^2^ in size, containing 4000 ppm of AITC) was attached to the Petri dish (90 mm diameter × 15 mm height) containing the eggs. The lid of the dish contained the AITC sheet, and the dish was sealed for 24 h. Essentially, the AITC sheet was not in direct contact with the eggs. The hatchability of the egg masses was measured after a month.

**Table 1:** Hatching rates of invasive flighted spongy moth complex eggs, whose hairy coverings had been removed, among treatments

	Control	Perm	Fenpr+Clo	AITC‐spray	AITC‐sheet
No. of hatch /200 eggs	148	0	0	0	0
Hatching rate (%)	74	0	0	0	0

Abbreviations: AITC, allyl isothiocyanate; fenpr+clo, fenpropathrin and clothianidin; perm, permethrin.

### 2.3 Acute toxicity test of AITC to FSMC eggs

Tests were conducted to determine the acute (24 h) toxicity of AITC on FSMC eggs. To adjust AITC concentration, a micropipette (NICHIRYO Inc., Saitama, Japan) was used, and the AITC reference standard (FUJIFILM WAKO PURE CHEMICAL Inc., 01‐0146, Saitama) was diluted with soybean oil (J‐OIL MILLS Inc., Tokyo, Japan). Three pairs of glass Petri dishes (46 mm diameter × 18 mm height) containing 10 mL of the different AITC solutions, adjusted to each test concentration, and 10 eggs of FSMC were placed in a desiccator (7 L volume) (DURAN, 200 mm; SIBATA SCIENTIFIC TECHNOLOGY Inc., Saitama, Japan). After 24 h, the eggs were removed from the desiccator and placed in plastic Petri dishes (55 mm diameter × 17 mm height), and then stored at 25 ± 1°C. The hatching ratio of FSMC was measured one month after the start of the experiments.

To understand the susceptibility of FSMC eggs to AITC, the nominal concentration of AITC was preliminarily tested in the range of 10 to 10 000 ppm (concentration ratio between successive solutions: 10). The formal test and adjusted methods for acute AITC toxicity were similar. The results showed susceptibility between 1000 and 10 000 ppm (29 eggs hatched at 1000 ppm, 4 eggs hatched at 10000 ppm). As the formal tests were conducted in the range of 1000–40 000 ppm (concentration ratio between successive solutions: 1.5–2.5), the concentrations were set as follows: 0 (soybean oil only), 1000, 2500, 5000, 10 000, 20 000, 30 000, 35 000, and 40 000 ppm.

A gas monitor (GX‐6000; RKI INSTRUMENTS Inc., Tokyo, Japan) was used to measure the concentration of volatile AITC in the desiccators. The gas monitor was set up using a volatile organic compound sensor with a sensitivity of <10.6 eV/ppm. The concentration that caused 50% hatching failure (i.e., lethal concentration: LC_50_) of FSMC was determined using a gas detector tube (No.149; measurement range of 5–200 ppm; Gastec Inc., Kanagawa, Japan) and then adjusted for the concentrations recorded by the gas monitor.

### 2.4 Statistical analysis

To detect the effects of each insecticidal treatment (control, AITC‐spray, AITC‐sheet, permethrin, and clothianidin) on the hatching rate of FSMC egg masses, a Kruskal–Wallis test was conducted. To test whether the hatching rates differed significantly among the treatments, a Steel–Dwass test was conducted.

The acute toxicity of AITC in FSMC eggs was corrected using the Abbott formula because of the need to correct for dead eggs in the controls. A generalized linear model (GLM) was used to investigate the relationship between corrected hatchability of FSMC after AITC exposure and gas concentration. The response variable was hatching rate, and the explanatory variable was the gas concentration of AITC (ppm). Explanatory variables were analyzed using an *F*‐test. When the hatching rate of FSMC sharply decreased, a logistic model was fitted, and a capacity‐response prediction curve and 95% confidence intervals were generated. Thus, the 50% lethal concentration (LC_50_), 90% lethal concentration (LC_90_), and standard error (SE) were calculated.

All statistical analyses were conducted using the statistical software R, version 4.4.1.^24^ The boxplot was generated using a package ‘ggplot2’.^25^ The *F*‐test was analyzed using package ‘car’.^26^ The LC_50_ and LC_90_ values and their SE were calculated using package ‘MASS’.^27^

## 3 RESULTS

### 3.1 Hatching rates of FSMC egg masses after exposure to AITC‐containing products and conventional insecticides

**Figure 1:**
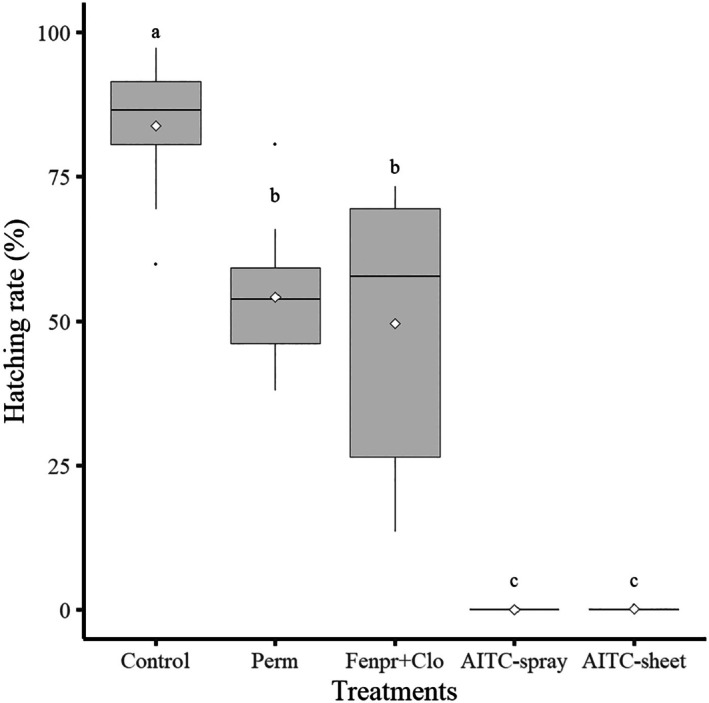
Box plot of the hatching rates of flighted spongy moth complex egg masses (*n* = 15) with different treatments: control, permethrin (perm), fenpropathrin and clothianidin (fenpr+clo), allyl isothiocyanate (AITC)‐spray, and AITC‐sheet. Open rhomboid (⋄) indicates the mean hatching rate (control: 84%; perm: 54%; fenpr+clo: 50%; AITC‐spray: 0%; AITC‐sheet: 2%). Different letters indicate significant differences (*P* < 0.05) among the five treatments according to the Steel–Dwass test.

There was a significant difference in the hatching rates of FSMC egg masses among the five treatments (*χ* ^2^ = 57.204, df = 4, *P* < 0.01, Kruskal–Wallis test). The mean hatchability of FSMC egg masses in the control group was 84%. For the two pyrethroid insecticide treatments, the mean hatching rates of the egg masses after permethrin (perm) and fenpropathrin and clothianidin (fenpr+clo) exposure were 50% and 54%, respectively, but their rates in the AITC treatments (AITC‐spray and AITC‐sheet) were nearly 0% (Fig. 1). The hatching rates of *L. dispar* egg masses after AITC treatment (spray/sheet) were significantly lower than those of the other treatments (*P* < 0.01, Steel–Dwass test). In contrast, no significant difference in hatchability was observed between the two AITC treatments (*P* > 0.05) (Fig. 1, Table 2).

**Table 2:** Differences in the mean hatching rates of flighted spongy moth complex egg masses across treatments

	*t*‐value	*P*‐value
Control: Perm	4.125	**
Control: Fenpr+Clo	3.455	**
Control: AITC‐spray	4.812	**
Control: AITC‐sheet	4.645	**
Perm: Fenpr+Clo	0.186	1 ^ns^
Perm: AITC‐spray	4.759	**
Perm: AITC‐sheet	4.572	**
Fenpr+Clo: AITC‐spray	4.576	**
Fenpr+Clo: AITC‐sheet	4.31	**
AITC‐spray: AITC‐sheet	1.797	0.38 ^ns^

*Note*: *P*‐value adjustment method: Steel–Dwass test. **: *P* < 0.01, ns: no significant.

Abbreviations: AITC, allyl isothiocyanate; fenpr+clo, fenpropathrin and clothianidin; perm, permethrin.

### 3.2 Lethal concentrations of AITC for FSMC eggs after gas treatment

**Figure 2:**
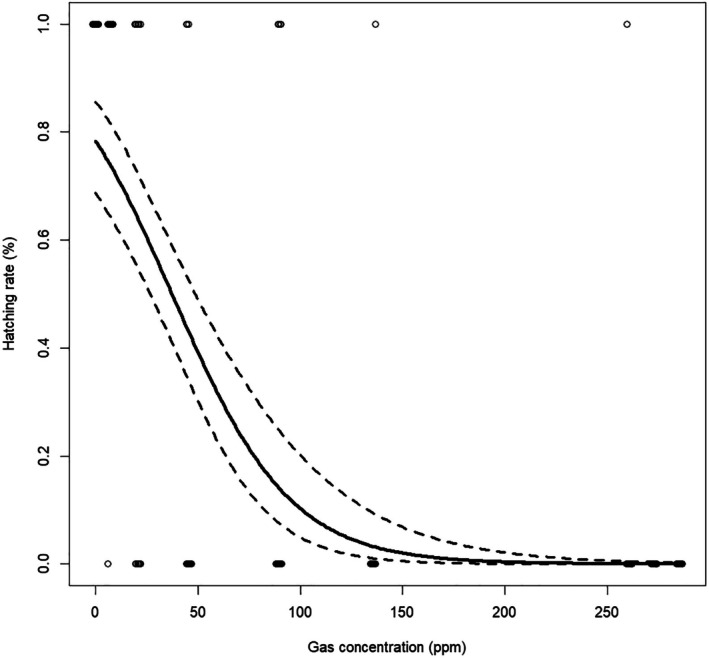
Predicted dose–response curves (solid line) of flighted spongy moth complex eggs to allyl isothiocyanate (AITC) gas concentration (ppm). Dashed black lines indicate the 95% confidence interval (CI) of the hatching rates, respectively.

**Figure 3:**
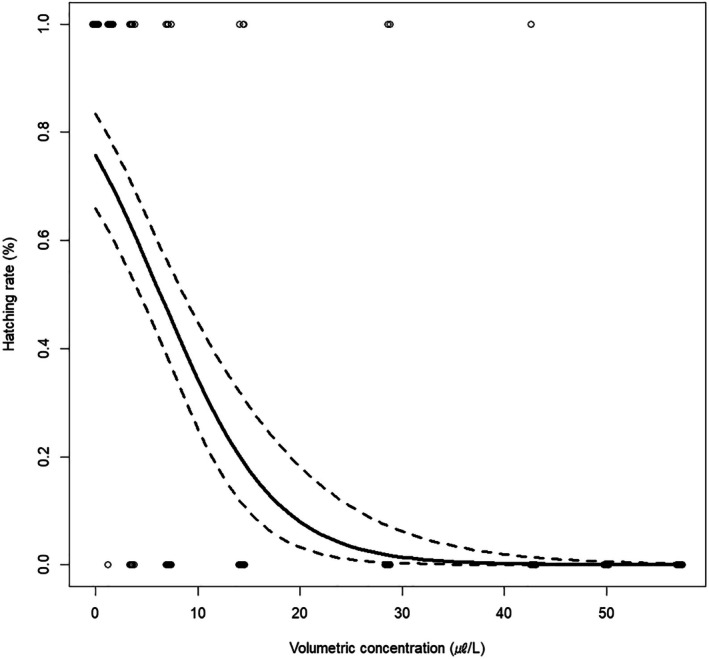
Predicted dose–response curves (solid line) of flighted spongy moth complex eggs to allyl isothiocyanate (AITC) concentration per volume (μL/L). Dashed black lines indicate the mean and 95% confidence interval (CI) of the hatching rates, respectively.

As the high efficacy of AITC in the hatching failure of FSMC egg masses was demonstrated, the detailed toxicity of the substance to the species was evaluated using an acute toxicity test. The concentrations of the AITC solutions, gas derived from them, adjusted volumetric concentrations, and corrected hatchability of FSMC are shown in Table 3. A significant decline in the egg hatching rate was observed with increasing AITC gas concentration (Fig. 2) and volumetric concentration (μL/L) (Fig. 3) (*P* < 0.01, GLM). The LC_50_ values of FSMC eggs to AITC were 37.3 ± 5.4 ppm in gas concentration and 6.4 ± 1.0 μL/L‐air per volume. Furthermore, its LC_90_ values were 100.9 ± 11.8 ppm in gas and 18.6 ± 2.5 μL/L‐air per volume (Table 4).

**Table 3:** Corrected hatch numbers of flighted spongy moth complex eggs 1 month after the exposure to allyl isothiocyanate solutions of different concentrations

Solution	Gas: ppm	Corrected hatch
(ppm)	(air: μL/L)	Number (*n =* 30)
0	0 (0)	30
1000	7.1 (1.4)	29
2500	20.5 (3.6)	12
5000	45.7 (7.1)	4
10 000	89.3 (14.3)	4
20 000	135.4 (28.6)	3
30 000	260.6 (42.9)	1
35 000	272.8 (50)	0
40 000	285.1 (57.1)	0

*Note*: Concentrations of allyl isothiocyanate solutions were converted into gas (ppm) and volumetric (μL/L) concentrations in a desiccator.

**Table 4:** Results for the generalized linear model and LC_50_ and LC_90_ of allyl isothiocyanate against flighted spongy moth complex eggs

	LC	
	μL/L	ppm
LC_50_ ± SE	6.4 ± 1.0	37.3 ± 5.4
LC_90_ ± SE	18.6 ± 2.5	100.9 ± 11.8
LC_99_ ± SE	32.0 ± 4.7	170.4 ± 22.0
*F*‐value	2.20E‐16	2.20E‐16
*z*‐value	4.647	5.051
*P*‐value	<0.0001	<0.0001

Abbreviations: LC, lethal concentration; SE, standard error.

## 4 DISCUSSION

In the present study, two distinct formulations (spray and sheet) containing AITC were used. For the spray‐type formulation, which contains AITC in soybean oil, it is reasonable to assume that the effects of both are exerted on FSMC egg masses upon spraying. This is because it has been reported that the eggs of FSMC were covered by spraying in a 20–30 cm radius around the egg masses with a soybean‐oil‐based insecticide.^17^ However, in our study, the AITC spray had a lethal effect on the eggs even though it could only wet the surface of the FSMC egg masses, and it is unlikely that the lethal effect on the eggs was the result of smothering by soybean oil rather than by the insecticidal action of AITC. One reason for this is that the hatching failure after the AITC‐sheet treatment was similar to that after AITC‐spray treatment. In addition, it is noteworthy that only AITC volatilized from the sheet affected egg masses. The extremely high toxicity (lethal effect) of the AITC‐containing product on FSMC egg masses/eggs was probably due to the passage of this highly volatile substance through the hairy covering on egg masses and subsequent interaction with the eggs. In contrast, the average hatching rates of FSMC egg masses with hairy covering after permethrin and clothianidin exposure were approximately 50% each, although both insecticides had an absolute lethal effect on eggs when the hairy covering of egg masses was removed (Fig. 1, Table 1). These results suggest that the hairy covering on the FSMC egg masses inhibited the penetration of both two insecticides (perm and fenpr+clo) into the eggs, which decreased their insecticidal efficacy.

The susceptibility of FSMC eggs to different AITC concentrations showed a significant reduction in the hatching rate with increasing AITC gas concentrations derived from its solutions (*P* < 0.05, GLM) (Table 4). The LC_50_ and LC_90_ values of the AITC gas concentration for FSMC eggs were 37.3 ± 5.4 ppm and 100.9 ± 11.8 ppm, respectively. These concentrations were 6.4 ± 1.0 μL/L‐air (LC_50_) and 18.6 ± 2.5 μL/L‐air (LC_90_) when converting the aforementioned values into concentration per volume (Table 4). Among insecticide treatments validating the hatching suppression of FSMC egg masses, the AITC‐spray treatment was conducted at 40000 ppm, which caused absolute hatching failure in the eggs. Incidentally, an AITC solution of 40 000 ppm corresponded to 285.1 ppm when converted to a gas concentration (Table 3). In addition, its gas concentration was above the eradication concentration (LC_99_: 32.0 ± 4.7 μL/L‐air, 170.4 ± 22.0 ppm) calculated based on the test for the acute toxicity of AITC to FSMC eggs. In contrast, the AITC sheet contained 4000 ppm AITC per 4 cm^2^, which was approximately equivalent to the gas concentration at the LC_50_ value of AITC for FSMC eggs. Therefore, it was concluded that the present gas concentration of the AITC‐sheet formulation could not achieve the absolute hatching failure of FSMC. Nonetheless, it should be noted that an experimental apparatus with different volumes was used for each test (i.e., differences in the hatching suppression of FSMC eggs among insecticides *versus* the acute toxicity of the species to AITC).

Limited knowledge exists regarding the susceptibility of eggs of other lepidopteran insects to AITC. However, its toxicity to their larvae has been evaluated, and the LC_50_ values of AITC for the third instar larvae of *Pieris rapae* Linnaeus, *Plutella xylostella* Linnaeus, and *Spodoptera litura* Fabricius were 2.0 μL/L‐air, 1.5 μL/L‐air, and 1.8 μL/L‐air, respectively.^28^ Our study revealed that the LC_50_ value of AITC for the eggs of *L. dispar* was 6.4 ± 1.0 μL/L‐air (Table 4). Comparing these data, the LC_50_ values for the larvae of the three species were less than one‐third of those for FSMC (i.e., high susceptibility), indicating that FSMC eggs may be significantly less susceptible to AITC among these Lepidoptera species. However, eggs and larvae were compared at different stages of development.

The LC_50_ values of AITC for *Tribolium castaneum* Herbst at each life stage^29^ were: 3.88 μL/L‐air for eggs (2.9 mm length and 0.7 mm width), 4.54 μL/L‐air for larvae (5–12 mm length and 2.1–3.7 mm width), 4.44 μL/L‐air for pupae (15.2 mm length and 3.8 mm width), and 4.42 μL/L‐air for adults (17.2 mm length and 4 mm width).^30^ This shows that the LC_50_ values of AITC for the egg stage of *T. castaneum* was lower (i.e., high susceptibility) than that for its other life stages. This suggests that the *T. castaneum* egg is the life stage most susceptible to insecticides. The circular eggs of *L. dispar* are 1.2 mm in diameter^31^ and smaller than those of *T. castaneum*.^30^ However, the LC_50_ value of FSMC eggs (6.4 ± 1.0 μL/L‐air) was higher (low susceptibility) than that of *T. castaneum* eggs (3.88 μL/L‐air), implying a weak correlation between the susceptibility of eggs to AITC and body size of species. This result contradicts previous findings (e.g.,^32^) that species sensitivity generally tends to decrease with increasing body size. Further studies are required on the responses of other arthropod eggs to AITC treatment to elucidate the factors that decrease egg susceptibility to this substance.

AITC is readily degraded in the atmosphere; thus, it is considered safe to both humans and the environment.^19^ Furthermore, as AITC is considered to have minimal persistence in exposed commodities,^33^ it can help to eradicate the unintentional introduction of FSMC *via* cargo to non‐native regions. In addition, our study revealed a clear insecticidal effect of AITC with high volatility against the eggs of FSMC regardless of the presence or absence of a hairy covering. Therefore, AITC may be an effective control method against arthropod eggs (e.g., *Latrodectus geometricus* egg sacs)^34^ with protective hair or silk coatings.

Our study revealed that AITC had lethal effects on FSMC egg masses with a thick hairy covering. In addition, a previous study revealed that fumigation with AITC had lethal effects on *S. invicta* in an enclosed space.^20^ Therefore, it is hypothesized that AITC may be used as a control method to simultaneously fumigate all life stages of arthropods, including the egg stage, when applied in an enclosed space.

## 5 CONCLUSION

Our results showed that AITC formulation with high volatility against the eggs of FSMC regardless of the presence or absence of a hairy covering. Furthermore, the susceptibility of FSMC eggs to different AITC concentrations was demonstrated. Differences in the susceptibility between FSMC and *T. castaneum* eggs to AITC contradicted the general reported tendency of chemical toxicity in relation to organism body size, suggesting that further studies on the response of other arthropod eggs to this substance are required. Fumigation with AITC may help prevent the unintentional transportation of invasive species, FSMC and other species, into non‐native regions *via* cargo in connection with trade.

## Data Availability

The data that support the findings of this study are available from the corresponding author upon reasonable request.
